# Deriving target exposure indices for common radiography exams based on automatic exposures of flat‐field phantoms

**DOI:** 10.1002/acm2.70562

**Published:** 2026-04-28

**Authors:** Matthew Hoerner, Emily L. Marshall, Katie Hulme, Ashley Tao, Suha Alshehri, Shady AlKhazzam, Zaiyang Long, Erin Macdonald, Kathleen Scilla, Ioannis Tsalafoutas

**Affiliations:** ^1^ Department of Radiology Yale New Haven Health New Haven, CT USA; ^2^ Department of Radiology University of Florida Gainesville, FL USA; ^3^ Department of Radiology Cleveland Clinic Cleveland, OH USA; ^4^ Department of Radiology Emplify Health by Gundersen La Crosse, WI USA; ^5^ Hamad Medical Corporation Doha Qatar; ^6^ Department of Radiology Mayo Clinic Rochester, MN USA; ^7^ Department of Radiology Duke University Durham, NC USA; ^8^ Associates in Medical Physics Akron, OH USA

**Keywords:** automatic exposure control, deviation index, digital radiography, quality control, radiography, target exposure index

## Abstract

**Background:**

The IEC exposure index (EI), deviation index (DI), and target exposure index (EI_T_), represent critical standardized metrics for the evaluation of exposure and quality in radiographic imaging.

**Purpose:**

This work develops and validates a systematic procedure to estimate the EI_T_ for eight of the most common radiography imaging protocols utilizing automatic exposure control (AEC) from measurements acquired under reference conditions.

**Methods:**

A model was developed to define the relationship between a systems AEC logic, and an estimation of the EI under flat‐field conditions (EIFFC). Separately, clinical data and acquisition protocol information for resultant EI during patient studies were also collected for the eight protocols studied: Chest posteroanterior (PA), Chest lateral, Abdomen anteroposterior (AP), Pelvis AP, L‐Spine AP, C‐Spine AP, T‐Spine AP, and Ribs AP. Data were collected from 41 x‐ray units spanning seven institutions. For each protocol on each unit the EIFFC was computed based on the acquisition protocol, as well as median EI from clinical exams to produce a scaling factor (SF). Kruskal–Wallis statistical tests were used to compare SF's between vendors and AEC cell configurations.

**Results:**

SFs for eight radiographic imaging protocols have been produced per vendor and per AEC cell selection. A workflow has been established for end‐users to follow to apply these SFs to flat‐field measurements taken at their own locations to establish local EI_T_.

**Conclusions:**

The study results show that in seven of the eight imaging protocols, the SFs for most units included in the study report SFs within 1 DI (± 25%) of their respective final vendor reported SF (218/228). The ribs protocol is the exception to this finding (n = 26). SFs have high utility for establishing EI_T_ values on individual x‐ray units and normalizing EI value distributions for quality assurance purposes.

## 1 INTRODUCTION

Digital radiography (DR) is the most utilized modality in Radiology departments across the United States when considering total number of acquired exams.^1^ As a result of the widespread use of this imaging modality, it is important that quality assurance measures are put in place and maintained to ensure clinical practice remains both safe and effective. DR based quality assurance programs commonly are made up of two components.^2^, ^3^ The first focuses on basic functions of the radiography unit and quantitative performance tests that ensure technical factors such as tube potential (kVp), tube loading (mAs), and accuracy of dose indications are within vendor or state provided criteria. The second component focuses on the qualitative performance of the system and the resultant images produced as determined by the radiologist.

Qualitative image performance evaluations reduce efficiencies in quality assurance programs as they are time intensive. With the introduction of digital image receptor exposure indicators, an opportunity presents itself to develop a clinically meaningful quantitative metric to facilitate the image assessment process. While these measures may not replace the need for specialist provided image assessment, they may reduce the burden of qualitative review by providing a quantitative metric to be used in aggregate for the consideration of institutional imaging data trends.

While in the past various implementations of exposure indicators were utilized by different vendors, a unified metric was introduced by the IEC applicable to new DR systems from all vendors in 2008 (IEC, 2008). The IEC exposure index (EI) is a metric used to assess exposure to the image receptor.^4^ This metric works conveniently as a tool for optimization in DR to reduce dose creep and limit the effects of electronic noise and quantum mottle.^5^, ^6^, ^7^, ^8^ When a DR system's automatic exposure control (AEC) mechanism is functioning as expected, every image of the same exam type, regardless of patient size, would be acquired with the same radiation level at the entrance surface of the image receptor. This corresponding exposure is defined as the target exposure index (EI_T_). Deviation from the EI_T_ is quantified by another IEC defined metric, the deviation index (DI). DI is a real‐time stand‐alone indicator providing technologists with immediate feedback on the acquisition technique, allowing them to adjust for any subsequent images. In this manner, DI serves as a tool for technologists to build their familiarity with imaging techniques and the resulting impact on image quality and diagnostic utility. Subsequently, DI may serve an important role in supporting technologists as they develop and refine manual technique charts for their own practice.

While the DI can serve as a real‐time feedback mechanism for technologists to understand the exposure condition of their acquired images, factors affecting DI must be understood before adoption into a clinical workstream. Further, the accuracy of the DI is limited based on the setting of an appropriate EI_T_. When an image is acquired with manual techniques, the resulting EI, and subsequent DI, rely primarily on the kVp and mAs set by the user. This relationship is clearly understood and enables users to make adjustments and improve EI and DI on subsequent exposures. When an image is instead acquired under AEC conditions, the relationship is no longer direct. AEC based images make use of calibration factors set by service at the time of unit installation with the potential for later adjustments during both routine and non‐routine service events. These calibration factors drive the mAs channel selection and ultimately result in the exposure level obtained at the image receptor directly leading to the resultant EI value. Changes in kVp or mAs may no longer result in a predictable change in DI. Achieving an appropriate EI_T_ level requires proper protocol settings and a well‐calibrated AEC system. At present, AEC techniques represent the gold standard of quality in non‐extremity adult digital radiographic imaging. As such, their resultant exposure indicators likely represent a desirable level of quality for non‐AEC based exams of the body. Even once this relationship is understood, it is rare that a system, even with EI_T_ values set to manufacture defaults, employing AEC techniques for clinical imaging, results in DI values at or near 0. While it is assumed by most clinical end‐users of the radiographic system that manufacturers have setup their manufacturer's EI_T_ values to be synchronous with their AEC calibration values for expected dose, the widespread DI values seen in most clinics demonstrates this is not the reality.^8^

The purpose of this work is to develop a methodology to estimate the EI_T_ for eight of the most common radiography imaging protocols that utilize AEC from measurements acquired under reference conditions. These methods do not require any form of retrospective data collection or patient dose trend input for end‐users.

## 2 METHODS

### 2.1 Exam selection for study inclusion

A selection of common radiography exam types was made utilizing the American College of Radiology (ACR) Dose Index Registry (DIR). Data extracted from the ACR DIR included all radiographic studies acquired from June 1, 2022 to MAy 1, 2023. Radiography acquisition data were then analyzed and restricted to include only those exam types that were taken under the use of AEC and reported a minimum of 10 000 cases. Upper and lower extremities were omitted from the study due to high occurrences of intra‐site variability in imaging acquisition parameters. The exam types with the highest volumes from the ACR DIR, which were then subsequently selected for trial, included the Chest posteroanterior (PA), Chest lateral, Abdomen anteroposterior (AP), Pelvis AP, L‐Spine AP, C‐Spine AP, T‐Spine AP, and Ribs AP.

### 2.2 Deriving scaling factors

**FIGURE 1:**
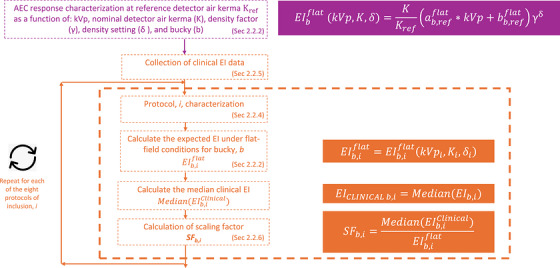
Workflow for the derivation of scaling factors.

The efforts outlined in this work focus on developing a methodology to estimate EI_T_ values for an x‐ray system without requiring any form of retrospective data collection or patient dose trend input for end‐users. This is accomplished by way of clinical system scaling factors (SFs) derived through data collected across multiple institutions and analyzed within this work. To develop this methodology, the EI reported by a DR system had to be benchmarked against a set of standardized reference conditions, and system‐specific radiography imaging techniques and clinical EI values had to be collected for patient images. These two components could then be combined to compute the ratio between clinical EI values and the expected EI under flat‐field conditions (EIFFC), referred to as the SF. At the completion of the process, a set of SFs were generated per exam type for end‐users to derive EI_T_ values from data that can be readily measured on their own system. The process associated with developing these SFs is outlined in Figure 1.

#### 2.2.1 Reference conditions for AEC characterization

This methodology is to model the AEC response and is not intended to evaluate AEC calibration. Each vendor calibrates their systems AEC differently, from attenuator differences to geometric setup differences, this information many times remains unavailable to the practicing physicist. There is an inherent assumption in this method that the system AEC has been calibrated to either manufacturer or institutional standards.

**FIGURE 2:**
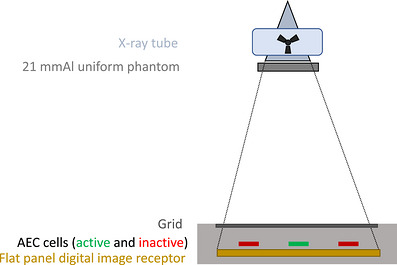
Illustration of reference conditions to characterize AEC parameters.

A set of fixed reference conditions was defined to measure the exposure incident on the image receptor under a homogenous (flat‐field) x‐ray field to establish a relationship between the AEC logic and the image receptor reported EI. Reference conditions included placing a 21‐mm aluminum filter (both alloy 1100 and 6061 were used dependent upon site) to fully intercept the x‐ray field, activating only the AEC central cell, kVp settings of 70, 100, and 120, and using the default speed setting (*S_ref_*) or detector air kerma (*K_ref_*) for the system without any density adjustment applied. Under these reference conditions, exposures were taken in both the wall and table bucky geometry configurations. Following each exposure, system reported mAs and EI (EI b, flat) were recorded. Figure 2 demonstrates the setup for reference condition image data acquisition. AEC response was then characterized based on a multi‐part model, the basis of which included a linear fit for EIFFC as a function of kVp. A worksheet for AEC data collection and characterization is provided in the online annex of this work.

#### 2.2.2 System AEC response model

The AEC response model took into consideration several acquisition parameters that determine exposure to the image receptor. Most AEC systems offer two parameters that directly adjust exposure to the image receptor: sensitivity and exposure correction. The metric of sensitivity in radiography has its roots in the early days of plain film, when specific anatomy required differences in film processing capacity on the basis of radiation exposure level. Sensitivity is defined using a speed class or dose level, typically fashioned in neighboring settings that are multiples of one another (e.g., 200, 400, 800). For the standard reference protocol, speed class *S_ref_*, is defined as:^9^

(1) $$S_{ref}=\frac{K_{0}}{K_{ref}}$$

where *K*_0_ = 0.001 Gy and *K_ref_* is the nominal air kerma at the image receptor under flat‐field conditions. When the speed class is adjusted from the standard reference protocol, the air kerma at the image receptor, *K_i_*, for protocol, *i*, with an updated speed class, *S_i_*, is defined as:

(2) $$K_{i}=K_{ref}\frac{S_{ref}}{S_{i}}$$

Exposure correction, sometimes referred to as density, is a parameter used to fine tune the detector exposure level (i.e., typically by steps of ± 15% or ± 25%). The exposure correction is expressed in terms of an exponentiation fit. The fit is defined as a fixed base equal to a derived value, defined in this work as the density factor (*γ*), raised to a power (*δ*) set on the system by an operator to a whole or half integer corresponding to the level of increase (positive) or decrease (negative) in air kerma. Equation (2) can therefore be expanded to include a density exposure adjustment, delta_*i*, for a given protocol:

(3) $$K_{i}\left(\delta_{i}\right)=K_{i}\gamma^{\delta_{i}}$$

It should be noted that for Equation (3), the density factor (*γ*) as proposed is derived using a log‐linear regression from measurements acquired of relative air kerma or EI at different density settings (*δ*) away from *K_ref_* at 70 kVp. The slope of such a regression would be equal to *γ*.

AEC systems are often calibrated to modify air kerma at the image receptor plane relative to tube potential to achieve a consistent detector dose, pixel value, signal‐difference‐to‐noise ratio or signal‐to‐noise‐ratio.^10^, ^11^, ^12^, ^13^ These relationships vary by vendor, and sometimes even more specifically by unit type and/or image receptor model. Therefore, a characterization step to account for the effect of kVp on image receptor air kerma was incorporated. This relationship was developed on the basis of the exposure index, as it is understood that this value maintains a direct relationship to the image receptor air kerma. These effects were defined using a linear fit, where a and c represented system‐specific fit‐parameters derived from image‐based measurements taken under the reference conditions described previously, where flat‐field exposures through a 21 mm Al filter were acquired at 70, 100, and 120 kVp in both bucky (*b*) geometries. Exposure index under reference conditions could then be as follows:

(4) $$El_{b}^{flat}\left(kVp\right)=a_{b,ref}^{flat}*kVp+c_{b,ref}^{flat}$$

The final model incorporates Equations ((2), (3), (4)). As previously discussed, the linear relationship between the EI and air kerma, when considering an x‐ray beam of fixed quality, allows for the substitution of air kerma in the place of EI. The reference air kerma at the image receptor, $K_{ref}$, represents the conditions under which the density factor and coefficients $a_{b,ref}^{flat}$ and $c_{b,ref}^{flat}$ were taken. Combining these dependencies into a single equation results in the following:

(5) $$El_{b,i}^{flat}\left(kVp_{i},K_{i},\delta_{i}\right)=\frac{K_{i}}{K_{ref}}\left(a_{b,ref}^{flat}*kVp_{i}+c_{b,ref}^{flat}\right)\gamma^{\delta_{i}}$$

Equation (5) defines the EIFFC when an image is acquired using the acquisition parameters for a clinical protocol, *i*, in bucky, *b*. This equation encapsulates the dependency of the reported EIFFC on the system AEC logic and permits the estimation of EIFFC for any set of acquisition parameters, provided the AEC has been characterized with the respective fit parameters.

#### 2.2.3 AEC configuration

The AEC contains an array of individual cells that sample different regions of the patient simultaneously during image acquisition. When the cell has detected sufficient signal (i.e., equal to $K_{ref}$), it will terminate an x‐ray exposure. Each cell may be individually selected to be inactive or active during the acquisition of an image.

During acquisitions taken under a manual technique option, variability in AEC cell response will be of no consequence, changing nothing about the image acquisition parameters nor the resultant image quality. When operating under AEC however, should a cell covered by highly attenuating anatomy be selected to drive the AEC logic, the selection will result in an increase in exposure time and an overall increase in exposure across the entire image. The reverse would be true in the event of selecting a cell covered by low attenuating anatomy, where we would expect a general reduction in exposure time and exposure across the entirety of the image. Cell selection in AEC acquired images has the potential to generate large variances in exposure output when imaging non‐uniform fields. Under the reference conditions, this cell dependence is irrelevant as the imaged object includes a flat‐field uniform phantom. Exceptions to this include non‐balanced AEC cells and grid cutoff. Under clinical conditions, however, the impact of AEC cell selection, as previously noted, could be significant. This impact would likely be dependent on the imaged body region. Therefore, SFs must be derived separately for each AEC cell configuration for a clinical protocol.

#### 2.2.4 Protocol characterization

When referring to the acquisition conditions defined for each clinical exam type, the term acquisition protocol is used. To compute $EI_{b,i}^{flat}$ using Equation (5), acquisition parameters for each of the eight clinical exam types were recorded from the system when setup in the clinical acquisition mode. The acquisition parameters included: AEC speed class or dose level, tube potential, exposure correction setting, bucky selection (table or wall), anti‐scatter grid, and active AEC cell selection. This protocol characterization step was repeated for every x‐ray unit that contributed data to this work.

#### 2.2.5 Collection of clinical EI data

Clinical EI image data were collected by exporting image acquisition logs from the x‐ray units, or the hospital dose monitoring systems (DMS), included in the study. The logs and DMS contain information about each image including the EI, protocol name, body part, view, and those acquisition parameters listed in 2.2.4. Once the data were exported, a filtering step was implemented on the parameters included in the logs, to limit the clinical data to include only those images acquired under the acquisition protocols as defined in Section 2.2.4 for the exam types under consideration. This step was essential to exclude cases where the default/official protocol had been manually altered by the radiographer, as this would alter the resultant EI. For each of the eight exams selected, the median EI was calculated from the filtered data. This clinical data collection step was repeated for every x‐ray unit that contributed data to this work. Lilliefors test was performed to test each distribution against a log‐linear model.

#### 2.2.6 Calculation of scaling factor

Ultimately, an SF was computed to link clinical EI values with EI values measured under flat‐field reference conditions. The objective of this SF was to create a simple way to derive EI_T_ values representative of typical clinical EI values from data that can be easily measured on a system, without having to collect large clinical data sets.

The SF is defined as the median ratio of the EI value from patient studies, as described in Section 2.2.5, for a given protocol, i, to the modeled EIFFC for identical acquisition parameters (Equation 5):

(6) $$\;SF_{b,i}=Median\;\left(\frac{EI_{b,i}^{clinical}}{EI_{b,i}^{flat}}\right)$$

For each of the x‐ray units evaluated, measurements were made at both the table and wall bucky to derive their respective fit parameters. When computing the SF for an individual exam type, the $EI_{b,i}^{flat}$ would be calculated using the bucky from which that specific protocol was acquired. SFs were computed per individual x‐ray system and exam type. This resulted in a total of 72 SFs computed as part of this work.

Once SFs were computed for each of the exam types under consideration, the values were consolidated across all systems included in the study. All x‐ray systems of the same vendor were grouped together, and acquisition protocols utilizing the same AEC cell configuration were further subdivided. The mean SF was then calculated per exam type for each vendor, with further subdivision for AEC cell configuration, to obtain a globally applicable SF.

### 2.3 Statistics associated with scaling factors

Statistical analysis was performed to evaluate units within the same vendor and exam type. Kruskal–Wallis testing was used to compare SFs for each unit against one another using a *p*‐value threshold of 0.01. A threshold of 1 DI was used to benchmark SFs from individual units against the vendor mean.

### 2.4 Application of scaling factors

**FIGURE 3:**
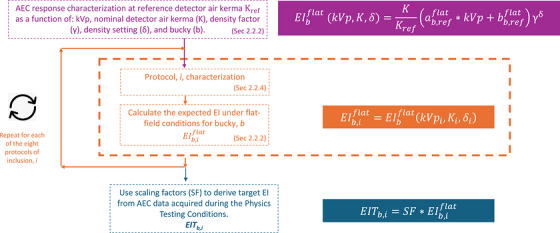
Workflow diagram outlining the process to be followed for end‐users to apply the final SFs to locally collected data.

Figure 3 outlines the process for end‐users to apply the SFs presented in this work, to their own AEC characterization data as described in Sections 2.2.1 and 2.2.2, to estimate EI_T_ values. The first step remains the same as described previously, AEC characterization must be completed under the predefined reference conditions. This can be done simply by following the worksheet provided in the online annex. The exam types on the system must then be considered, and protocols defined to include acquisition parameter values for bucky, kVp, nominal detector air kerma, and density. The EI expected under flat‐field conditions for each protocol's acquisition parameters can then be estimated using Equation (5). This value can then be multiplied by the appropriate SF produced in this work to derive an EI_T_. Equation (7) highlights the estimated EI_T_ value for end‐users based on this process.

(7) $$EIT_{b,i}=SF_{b,i}\times EI_{b,i}^{flat}$$

## 3 RESULTS

To develop the SFs, data were collected from seven institutions with various equipment vendors. Table 1 shows the total number of institutions, x‐ray systems, and clinical studies that contributed data towards the estimation of the final SFs for each exam type by vendor. SFs were computed for every clinical image included in the study for evaluation. Once values were computed for all clinical images on a single unit, the median SF was computed for that specific unit per individual exam type (Equation 6). The mean SFs for each vendor were determined per AEC configuration and exam type. These are the SF values reported in Table 2.

**TABLE 1:** The total number of institutions, x‐ray units, and clinical exams that contributed data towards the estimation of the final scaling factors for each exam type by vendor. Differences in AEC cell configuration resulted in different scaling factors, thus contributing data is subdivided by AEC cell configuration in column one as well.

	Number of institutions/number of units/number of exams by manufacturer							
Exam Type (Body Region, View, AEC Config)	Carestream	Fuji	GE	Konica	Philips	Samsung	Canon	Siemens
Chest PA—LR	1 / 2 / 2004	1 / 1 / 1696	4 / 10 / 5679	1 / 2 / 563	1 / 4 / 3762	1 / 1 / 1267	1 / 5 / 1698	2 / 2 / 4043
Chest PA—LC	0 / 0 / 0	1 / 2 / 2328	0 / 0 / 0	0 / 0 / 0	0 / 0 / 0	1 / 1 / 262	0 / 0 / 0	1 / 2 / 337
Chest Lat—C	1 / 3 / 1737	1 / 3 / 4117	3 / 5 / 2506	1 / 2 / 525	2 / 6 / 14611	1 / 2 / 1471	1 / 5 / 1671	2 / 4 / 6413
Abdomen AP—LCR	1 / 2 / 164	0 / 0 / 0	3 / 8 / 1189	0 / 0 / 0	0 / 0 / 0	1 / 2 / 456	1 / 1 / 52	1 / 3 / 646
Abdomen AP—LR	1 / 1 / 42	1 / 3 / 99	0 / 0 / 0	0 / 0 / 0	2 / 6 / 1752	0 / 0 / 0	1 / 3 / 336	0 / 0 / 0
Abdomen AP—C	0 / 0 / 0	0 / 0 / 0	2 / 3 / 426	1 / 2 / 302	1 / 2 / 209	0 / 0 / 0	0 / 0 / 0	1 / 2 / 873
Pelvis AP—LR	1 / 3 / 329	1 / 2 / 230	3 / 8 / 666	0 / 0 / 0	2 / 6 / 244	1 / 2 / 681	1 / 4 / 1250	1 / 3 / 440
L‐Spine AP—C	1 / 3 / 470	1 / 3 / 273	4 / 10 / 1410	0 / 0 / 0	2 / 7 / 253	1 / 2 / 398	1 / 5 / 414	2 / 5 / 2536
C‐Spine AP—C	1 / 2 / 261	1 / 1 / 84	4 / 10 / 1298	1 / 2 / 67	2 / 9 / 438	1 / 2 / 328	1 / 5 / 199	2 / 5 / 1532
T‐Spine AP—C	1 / 3 / 190	1 / 3 / 53	4 / 10 / 313	0 / 0 / 0	2 / 7 / 89	1 / 2 / 96	1 / 5 / 143	2 / 5 / 508
Ribs AP—C	1 / 3 / 158	1 / 1 / 91	3 / 7 / 215	1 / 1 / 41	2 / 4 / 77	1 / 1 / 14	1 / 1 / 11	2 / 5 / 118
Ribs AP—LR	0 / 0 / 0	1 / 2 / 51	0 / 0 / 0	0 / 0 / 0	0 / 0 / 0	1 / 1 / 159	0 / 0 / 0	0 / 0 / 0

Abbreviations: C, center AEC cell; L, left AEC cell; R, right AEC cell.

**TABLE 2:** The mean scaling factors for each vendor per AEC configuration and acquisition protocol.

Exam Type (Body Region, View, AEC Config)	Scaling factor								% within Protocol Mean
	Carestream	Fuji	GE	Konica	Philips	Samsung	Canon	Siemens	
Chest PA—LR	0.37	0.39	0.37	0.35	1.02	0.31	0.29	0.38	63%
Chest PA—LC	–	0.39	–	–	–	0.42	–	0.58	67%
Chest Lat—C	0.69	0.37	0.58	0.62	1.14	0.55	0.58	0.67	75%
Abdomen AP—LCR	1.13	–	0.87	–	–	0.70	–	1.08	40%
Abdomen AP—LR	1.38	0.98	–	–	0.78	0.70	0.71	–	40%
Abdomen AP—C	–	–	1.27	0.71	0.94	–	–	1.38	50%
Pelvis AP—LR	1.24	1.70	1.14	0.68	0.95	0.98	0.93	1.49	63%
L‐Spine AP—C	1.03	0.95	1.04	–	0.80	0.93	0.91	1.06	100%
C‐Spine AP—C	1.02	0.87	0.45	0.48	0.57	0.90	0.87	0.77	50%
T‐Spine AP—C	1.89	1.10	1.21	–	0.57	1.26	1.01	1.13	71%
Ribs AP—C	0.92	–	0.89	0.22	3.67	0.74	1.11	0.85	14%
Ribs AP—LR	–	0.46	–	–	–	0.66	–	–	–

Abbreviations: C, center AEC cell; L, left AEC cell; R, right AEC cell.

**FIGURE 4:**
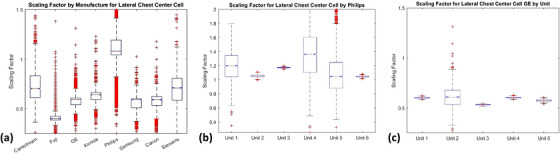
Box and Whisker plots of scaling factor for lateral chest protocol using the center cell by (a) vendor (b) Philips (c) GE. Box plots graphically display the upper quartile and lower quartile, in blue, the median, in red, the 1.5× interquartile range, in black, and outliers (outside the 1.5× interquartile range), as red cross marks.

Calculated SFs for all lateral chest protocol clinical images are shown in Figure 4. While most of the vendors for this protocol demonstrate comparable estimations of SF, with median values centered around 0.65, the graph demonstrates a clear difference in the Philips resultant SF when compared to the other vendors. While the overall median for Philips differs from the other vendors, when comparing the individual units that contributed data to the calculation of the Philips SF, the values remain consistent (Figure 4b). All six of the systems individual SF fall within 25% of the Philips lateral chest SF of 1.14. In addition to the Philips data, Figure 4c illustrates the clinical cases that made up the GE SF. Again, all five of the systems, taken from three different institutions, individual SFs fall within 25% of the GE lateral chest SF of 0.58. These results demonstrate intra‐vendor consistency between resultant SF and the statistical trends found within the clinical data collected.

Due to the variability in SFs between vendors for individual protocols, it was decided to produce vendor specific SFs as opposed to aggregating all vendors into a single value for estimation per protocol. The trends demonstrated in Figure 4 highlight the statistically significant differences seen between vendors, while also providing assurances that within any single vendor, the mean SFs offer statistically representative values for the cases from that individual vendor. When specifically considering the lateral chest data, six of the eight vendors included resulted in an SF within 25% (1 DI) of the mean value across all vendors (Mean SF_lateral chest: 0.651).

**FIGURE 5:**
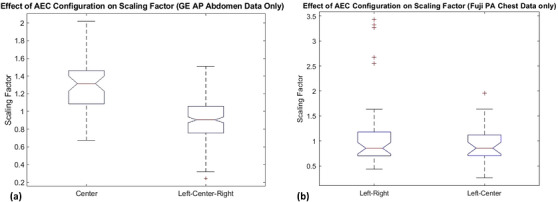
Effect of AEC configuration on scaling factor for (a) GE AP abdomen protocols for center (C) and left‐center‐right (LCR) cell selections, and (b) Fuji PA chest protocols for left‐right (LR) and left‐center (LC).

The impact of AEC cell selection was explored by considering the SFs resulting from those protocol/vendor combinations found within the contributed clinical data that utilized more than one AEC cell selection. Both the chest PA protocol, and the abdomen AP protocol, had individual vendors with two AEC cell selections in the clinical datasets. The chest PA protocol had data collected for three vendors with two different AEC cell selections (left/right and left/center). The abdomen AP protocol had data collected for six vendors with three different AEC cell selections (left/center/right, left/right, and center). Figure 5a shows the abdomen AP protocol clinical case SFs for GE separated out by AEC cell selection. The GE data collected had clinical cases with both left/center/right (SF_Abdomen AP, GE, LCR: 0.87) and center only (SF_Abdomen AP, GE, center: 1.27) cell selection. The difference between these two datasets is statistically significant (*p* < 0.01). Figure 5b shows the chest PA protocol clinical case SFs for Fuji separated out by AEC cell selection. The Fuji data collected had clinical cases with both left/right and left/center (SF for both configurations: 0.39). The differences between these two datasets were not statistically significant (*p* = 0.89)

**FIGURE 6:**
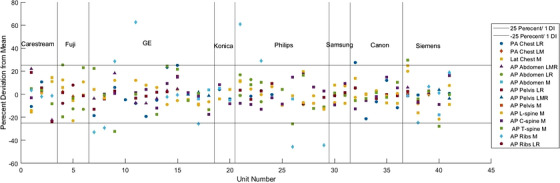
Scatter plot of all scaling factors plotted relative to their vendor mean for every unit and acquisition protocol. Units are grouped by vendor using vertical lines. The two horizontal lines represent ± 1 deviation index from the mean.

Figure 6 provides a graphical representation of the SF deviations from the protocol mean, for every unit included in this study. Percent differences were evaluated by taking the difference between an individual unit's SF and that of the mean SF calculated for its specific vendor (SF_vendor) for the given protocol, divided by the SF_vendor. Not all units contributed data for each of the eight clinical protocols. Each of the units is represented by an individual point on the x‐axis of the figure. Their individual SF's deviation from vendor mean are represented with various points along the y‐axis, with graphical variations depending on exam type. This figure highlights the tight fit of almost all units tested to the vendor calculated mean SF (i.e., SF_vendor, values as presented in Table 2). Only 20 of the 249 data points plotted fall outside of the indicated 25% difference line between vendor SF and unit SF for an individual exam type.

Table 3 summarizes the statistical results for unit‐by‐unit comparisons within the same vendor and the same exam type. The table shows the number of matches, defined as the *p*‐value greater than 0.01, the number of possible matches, and the percentage of matches. In most instances, the units had statistically different SFs, however, Table 4 shows that most units had SFs within ± 1 DI of the mean SF. This table demonstrates better agreement, as almost all acquisition protocols, excluding the ribs and t‐spine, showed over 90% of systems reporting agreement to within 1 DI of the vendor SF.

**TABLE 3:** Statistical results for unit‐by‐unit comparisons within the same vendor and exam type.

Exam Type (Body Region, View, AEC Config)	Unit SF, *p* > 0.01 (instances, possible matches, % match)								
	Carestream	Fuji	GE	Konica	Philips	Samsung	Canon	Siemens	All
Chest PA—LR	0 / 1 / 0%	–	4 / 45 / 8%	0 / 1 / 0%	0 / 6 / 0%	–	0 / 10 / 0%	0 / 1 / 0%	4 / 64 / 6%
Chest PA—LC	–	0 / 1 / 0%	–	–	–	–	–	0 / 1 / 0%	0 / 2 / 0%
Chest Lat—C	0 / 3 / 0%	1 / 3 / 33%	1 / 10 / 10%	0 / 1 / 0%	0 / 15 / 0%	0 / 1 / 0%	2 / 10 / 10%	0 / 6 / 0%	3 / 49 / 6%
Abdomen AP—LCR	0 / 1 / 0%	–	2 / 28 / 7%	–	–	1 / 1 / 100%	–	0 / 3 / 0%	3 / 33 / 9%
Abdomen AP—LR	–	1 / 3 / 33%	–	–	2 / 15 / 13%	–	0 / 3 / 0%	–	3 / 21 / 14%
Abdomen AP—C	–	–	0 / 3 / 0%	0 / 1 / 0%	0 / 1 / 0%	–	–	0 / 1 / 0%	0 / 6 / 0%
Pelvis AP—LR	0 / 3 / 0%	1 / 1 / 100%	6 / 28 / 21%	–	4 / 15 / 26%	0 / 1 / 0%	1 / 6 / 16%	0 / 3 / 0%	12 / 57 / 21%
L‐Spine AP—C	0 / 3 / 0%	1 / 3 / 33%	6 / 45 / 13%	–	7 / 21 / 33%	0 / 1 / 0%	2 / 10 / 20%	0 / 10 / 0%	16 / 93 / 17%
C‐Spine AP—C	0 / 1 / 0%	–	9 / 45 / 20%	0 / 1 / 0%	5 / 36 / 13%	0 / 1 / 0%	2 / 10 / 20%	0 / 10 / 0%	16 / 104 / 15%
T‐Spine AP—C	1 / 3 / 33%	1 / 3 / 33%	11 / 45 / 24%	–	12 / 21 / 57%	1 / 1 / 100%	5 / 10 / 50%	2 / 10 / 20%	33 / 93 / 35%
Ribs AP—C	1 / 3 / 33%	–	8 / 21 / 38%	–	1 / 6 / 16%	–	–	3 / 10 / 30%	13 / 40 / 32%
Ribs AP—LR	–	0 / 1 / 0%	–	–	–	–	–	–	0 / 1 / 0%

Abbreviations: C, center AEC cell; L, left AEC cell; R, right AEC cell.

**TABLE 4:** Comparison of individual unit to the vendor mean scaling factor. Evaluation of the percentage of units scaling factors’ that are within ± 1 deviation index of the vendor mean scaling factor.

Exam Type (Body Region, View, AEC Config)	Unit SF within ± 1 DI of manufacture mean (instances, possible matches, % match)								
	Carestream	Fuji	GE	Konica	Philips	Samsung	Canon	Siemens	All
Chest PA—LR	2 / 2 / 100%	–	10 / 10 / 100%	2 / 2 / 100%	4 / 4 / 100%	–	3 / 5 / 60%	2 / 2 / 100%	23 / 25 / 92%
Chest PA—LC	–	2 / 2 / 100%	–	–	–	–	–	2 / 2 / 100%	4 / 4 / 100%
Chest Lat—C	3 / 3 / 100%	3 / 3 / 100%	5 / 5 / 100%	2 / 2 / 100%	6 / 6 / 100%	2 / 2 / 100%	5 / 5 / 100%	4 / 4 / 100%	30 / 30 / 100%
Abdomen AP—LCR	1 / 2 / 50%	–	8 / 8 / 100%	–	–	2 / 2 / 100%	–	3 / 3 / 100%	14 / 15 / 93%
Abdomen AP—LR	–	3 / 3 / 100%	–	–	6 / 6 / 100%	–	3 / 3 / 100%	–	12 / 12 / 100%
Abdomen AP—C	–	–	3 / 3 / 100%	2 / 2 / 100%	2 / 2 / 100%	–	–	2 / 2 / 100%	9 / 9 / 100%
Pelvis AP—LR	2 / 3 / 66%	2 / 2 / 100%	8 / 8 / 100%	–	6 / 6 / 100%	2 / 2 / 100%	4 / 4 / 100%	3 / 3 / 100%	27 / 28 / 96%
L‐Spine AP—C	3 / 3 / 100%	2 / 3 / 66%	10 / 10 / 100%	–	7 / 7 / 100%	2 / 2 / 100%	5 / 5 / 100%	4 / 5 / 80%	33 / 35 / 94%
C‐Spine AP—C	2 / 2 / 100%	–	10 / 10 / 100%	2 / 2 / 100%	9 / 9 / 100%	2 / 2 / 100%	5 / 5 / 100%	5 / 5 / 100%	35 / 35 / 100%
T‐Spine AP—C	3 / 3 / 100%	3 / 3 / 100%	9 / 10 / 90%	–	6 / 7 / 85%	2 / 2 / 100%	5 / 5 / 100%	3 / 5 / 60%	31 / 35 / 88%
Ribs AP—C	3 / 3 / 100%	–	2 / 7 / 28%	–	0 / 4 / 0%	–	–	4 / 5 / 80%	9 / 19 / 47%
Ribs AP—LR	–	2 / 2 / 100%	–	–	–	–	–	–	2 / 2 / 100%

Abbreviations: C, center AEC cell; L, left AEC cell; R, right AEC cell.

## 4 DISCUSSION

This methodology does not optimize image quality nor radiation dose, it instead reflects the systems AEC response and its utilization in current clinical practice. Should a site be interested in incorporating image quality review into EI_T_ management, a radiologist should be consulted and image reader studies performed to select appropriate techniques and anticipated EI values. This method is intended instead to support the clinical medical physicist in their acceptance testing of new equipment, when image statistics are not yet available, and in adjustment of EI_T_ values to match current practice when image statistic review is unfeasible. The establishment of appropriate EI_T_ values serves as a critical first step in DI utilization for clinical exams. When an exam's target values and nominal detector air kerma are discordant, DI readings will misrepresent the patient dose condition – suggesting under‐ or over‐exposure when the technique was actually appropriate.

The data establishes that the EI generated under a defined flat‐field condition is not equivalent to the EI reported for clinical examinations. This would be consistent with the IEC definition of the EI, which provides vendors latitude to define their own region of interest (ROI) for the selecting of imaged pixels used to calculate the EI. Variability in vendor implementation results in large differences seen in calculated EI for set anatomical exams. This effect was particularly noted for body regions that contain substantial portions of air and bone, like the chest and pelvis. The effect of EI calculation is shown to be highly dependent upon the method that the vendors have individually chosen to define the clinically relevant imaging ROI. Figure 4 highlights this effect, as the estimated lateral chest SF for Philips demonstrates an obvious difference when compared against the other vendors. Philips uses an anatomical ROI to determine EI (lung field), which contains higher exposed pixels. This stands in contrast to other vendors who may instead utilize a mean or median pixel value taken across the entirety of the acquired image, thus including the mediastinum and diaphragm as well as tissues above the lung apices. When engaging both the right and left AEC cells during a PA chest acquisition on a Philips DR system, the exposure control region and image exposure region overlap significantly. This results in an SF close to 1. The presence of bone, air, and tissue adds complexity to the ROI determination for the EI. This results in significant deviations from unity when the AEC configuration (i,e, exposure control region) is placed behind one tissue type while the ROI (i.e., image exposure region) is defined within another.

It was difficult to assess the effect of AEC cell selection due to the lack of variation in configurations for the same body region and vendor. To most appropriately consider the variability of AEC cell selection, clinical data would need to be collected for a single vendor, under a single protocol, but with variable AEC cell selections. Across all sites’ clinical data collection, this variability only occurred in two clinical protocols, the Chest PA (with three vendors) and the Abdomen AP (with five vendors). The manner in which the AEC itself operates is also a factor. Some AEC systems are configured to collect cumulative exposure values across all active cells and average this signal to compare against an expected reference signal. The effect of AEC cell selection in Figure 5 shows how inclusion of the center cell, which is obstructed by the spine, can increase detector exposure. Alternatively, the AEC circuitry may be configured to compare the exposure values accumulated by individual cells to the expected reference signal, to determine cutoff time. Table 2 shows this effect for Fuji Chest. Both AEC cell selections collected in the clinical data were acquired using the left cell. The similarities in their resultant SFs, across both variations in cell selections, appear to be based solely on their use of the left cell, and its likelihood to terminate first. When imaging the PA chest, the left cell is not obstructed by the myocardium, so it will be exposed the fastest and drive the AEC logic on this exposure. While differences in vendor AEC logic are one contributor to resultant image quality; institutional standards and attentive protocol management serve as another. An understanding of how a specific unit's AEC logic functions, and the processes for patient positioning associated with a specific exam, allows a team to locally evaluate AEC cell selection to target specific image quality standards.

SFs were consistent from unit to unit, as demonstrated in Table 4 when using 1 DI as a benchmark. The most significant exception to this was the AP ribs. Different facilities acquire ribs differently by separating imaging protocols into upper and lower acquisitions and laterality. The selection of the center cell, which likely lies below the spine with the lungs on either side, presents a high spatial gradient in x‐ray attenuation. This renders the image exposure determination more susceptible to alterations in patient positioning when compared to the more anatomically uniform abdominal region. The thoracic spine acquisition protocol had the second lowest rate of consistency at 88%.

There are several notable limitations to this study. The attenuator chosen for the reference condition, aluminum, placed at the x‐ray tube port models the spectral composition of x‐rays transversing a patient. This attenuator, however, does not adequately simulate clinical scatter properties. The effect of scatter is dependent on patient habitus, so any attenuator would be susceptible to this effect. There could be a dependence on bucky system design, which affects how the AEC terminates exposure under scatter conditions with respect to how the detector intercepts the scattered x‐rays. Aluminum was chosen because of its lightweight properties and widespread use in other areas of x‐ray system evaluation, including AEC calibration, detector calibration, and exposure index accuracy. An evaluation and comparison between phantoms made up of copper and aluminum was made, the results showed similarities in resultant spectra, leading to a minimum impact on AEC response. The second limitation of this study is not accounting for cell balance, which may play a key role in variations between units. The impact an imbalance of AEC cells may have on this method has not been quantified. Though an instance where a single selected lateral cell is off by 20% and requires exposure time to be lengthened accordingly, it is expected that this too would result in a displayed EI on the system 20% higher than expected. The third limitation is that we did not account for AEC response time under clinical conditions (small patients) or under reference conditions (imaging with the flat‐field aluminum phantom). The fourth limitation was that the clinical conditions were not reproducible. There was variability in patient positioning with respect to the AEC cell selection, collimated fields of view, and detector orientation (landscape vs. portrait). An additional consideration that may limit the calculated SFs is the size variability of the patients imaged at each site. While a well‐established AEC system would maintain exposure consistency at the image receptor regardless of patient size, there would be an expected impact from variable thicknesses on scatter conditions. The EIs resulting from patient images were used for the estimation of SFs without controlling for patient size. Overall, this was intentional as the methodology was intended to simplify a complex relationship, providing a workflow to any clinical physicist for incorporation into their quality assurance program.

The final limitation considers the variability and lack of comprehensive x‐ray acquisition data provided from systems. Clinical data were extracted from all units included in the study via image acquisition logs provided by the vendors, or DMS. There was a high degree of variability in the exposure log information available for filtering between vendors. Some vendors produced the logs that included every acquisition parameter, including AEC cell selection, AEC dose level and speed, kVp, bucky, and acquisition protocol. Others omitted even the most basic parameters, such as bucky and kVp.

Researchers at each institution were tasked with filtering their clinical acquisition data to only include those images that were acquired under their standard protocol. This step was limited when information was omitted from clinical exposure logs. This may result in a variation in SFs estimated for those vendors that provided less information in their exposure logs.

## 5 CONCLUSION

This study presents a methodology to estimate clinical EI_T_ values for acquisition protocols that involve an AEC system, and presents vendor‐specific SFs derived for eight common exam types. This study establishes the dependence of clinical EI values on acquisition protocol and vendor algorithm. SFs have high utility for establishing target EI values on individual x‐ray units and normalizing EI value distributions for comparison purposes, such as the establishment of a diagnostic reference level.

## AUTHOR CONTRIBUTIONS

Authors E.L.M., M.H., K.H., and A.T. developed the methodology evaluated in the work. All authors contributed data to validate the methods. E.L.M. managed the IRB and data deidentification and storage. M.H. processed all data. M.H. and E.L.M. drafted the manuscript. Editorial feedback was provided by all authorship.

## CONFLICT OF INTEREST STATEMENT

The authors declare no conflicts of interest.

## ETHICS STATEMENT

This work required the collection of retrospective radiographic imaging data to be exported and shared across institutions for data aggregation and statistical analysis. These efforts were managed under the University of Florida IRB protocol #IRB202302012. All data contributing sites submitted for, and obtained, local institutional IRB and managed data collection and sharing in line with their own individual respective requirements.
